# Etiological workup of pseudotumor cerebri in pediatric patients—is it really necessary?

**DOI:** 10.1007/s00431-025-06389-x

**Published:** 2025-09-15

**Authors:** Lotem Goldberg, Gad Dotan, Nina Shirman Erel, Hila Weisblum Neuman, Keren Smuel Zilberberg, Danielle Kadishevich, Yehonatan Pasternak, Idan Goldberg, Yoel Levinsky, Dror Kraus, Oded Scheuerman

**Affiliations:** 1 Department of Pediatrics B, Schneider Childrens Medical Center of Israel, Petah Tiqva, Israel; 2 Gray Faculty of Medical and Health Science, Tel Aviv University, Tel Aviv, Israel; 3 Institute of Pediatric Ophthalmology, Schneider Childrens Medical Center of Israel, Petah Tiqva, Israel; 4 Department of Pediatrics A, Schneider Childrens Medical Center of Israel, Petah Tiqva, Israel; 5 Institute of Pediatric Neurology, Schneider Childrens Medical Center of Israel, Petah Tiqva, Israel; 6 Institute of Hematology, Rabin Medical Center, Petah Tiqva, Israel

**Keywords:** Pseudotumor cerebri, Intracranial hypertension, Headache, Secondary causes, Diagnostic workup

## Abstract

Pseudotumor cerebri (PTC) is a relatively common cause of headaches in children. In order to exclude secondary causes, an extensive laboratory workup is generally recommended. This assessment causes blood loss, discomfort and a financial burden. To our knowledge, the diagnostic significance of such workup is unclear. We aimed to evaluate the clinical yield of PTC laboratory workup in a pediatric tertiary medical center**. **This is a retrospective study in a tertiary pediatric hospital. Included were children hospitalized between 2010–2020 with new diagnosis of definite PTC, as confirmed by a certified pediatrician and ophthalmologist. Abnormal results were reviewed by two pediatricians for clinical significance. 75 children (58.7% girls, mean age 10.9 ± 4.25 years) with PTC were included. Secondary known causes for PTC were found in 20%, and 28% had other pre-existing medical conditions. Mean BMI was 24.53 ± 8.65 kg/m^2^. Vitamin D insufficiency (< 50 nmol/L) was diagnosed in 68%, with over half with deficiency (less than 30 nmol/L). Other than being overweight, the most common identifiable etiologies of PTC were drug-related. No additional secondary cases were diagnosed due to the extended work-up.

*Conclusion*: Our findings suggest that most PTC etiologies can be identified through medical history and physical examination, which may imply that an extensive laboratory work-up, except for vitamin D levels, may not be routinely required.

**Table:** 

**What is known:** • *An extensive laboratory workup is routinely recommended to exclude secondary PTC.* • *The clinical utility of PTC workup has not been assessed.*
**What is new:** • *A comprehensive medical history and physical examination probably suffices to diagnose secondary PTC.* • *An extensive laboratory work-up, except for vitamin D levels, may not be routinely required.*

**What is known:**

• *An extensive laboratory workup is routinely recommended to exclude secondary PTC.*

• *The clinical utility of PTC workup has not been assessed.*

**What is new:**

• *A comprehensive medical history and physical examination probably suffices to diagnose secondary PTC.*

• *An extensive laboratory work-up, except for vitamin D levels, may not be routinely required.*

## Introduction

Pseudotumor cerebri (PTC), also known as idiopathic intracranial hypertension (IIH), is a neurological syndrome characterized by signs and symptoms of elevated intracranial pressure (ICP), with normal brain parenchyma and cerebrospinal fluid (CSF) composition [1, 2]. The syndrome is diagnosed more commonly in obese young women, but can also affect children and adults of normal weight. PTC is most commonly a primary disorder. When PTC symptoms are attributed to an identifiable cause, excluding overweight body habitus, the condition is classified as secondary PTC [1–5]. Pediatric PTC has an estimated annual incidence of 0.5–0.9 per 100,000, and up to 3.5 per 100,000 in women aged 15–44 years [6, 7]. In recent years, the global obesity epidemic has led to an increase in the incidence of PTC [4, 8].

The modified Dandy criteria for the diagnosis of PTC requires signs or symptoms of increased ICP, a normal neurological examination (except abducens nerve palsy), increased lumbar puncture (LP) opening pressure with normal CSF composition, and absence of a space occupying lesion causing elevated ICP on brain imaging [3, 9]. Friedman et al. revised these criteria, so a PTC diagnosis could be made in the absence of papilledema if there is abducens nerve palsy and neuroimaging criteria suggestive of increased ICP. An elevated opening pressure is defined above 25 cm CSF (or 28 cm CSF if the child is sedated or obese) [7].

Current recommendations for children, elderly, males or non-obese females diagnosed with PTC are to undergo a wide diagnostic investigation for possible underlying causes [1, 10, 11]. According to the current literature, a secondary cause other than obesity is found in 21%−45% of pediatric PTC cases. Common etiologies include medication use (mainly tetracycline antibiotics, isotretinoin, hormonal treatment, glucocorticoid treatment or withdrawal, oral contraceptive pills, etc.), endocrine abnormalities, chronic kidney diseases and sino-pulmonary infections [12, 13]. Etiologies unique in the pediatric population include growth hormone replacement therapy and acne medications. Accordingly, the etiological work-up for PTC includes renal, liver, thyroid, hematological, inflammatory, and autoimmune blood profiles [12, 14].

Mollan et al*.* developed multidisciplinary guidelines for the management and investigation of PTC. They suggested that when patients are atypical (not female at childbearing ages or Body mass index (BMI) < 30 kg/m^2^), additional tests may be considered to exclude secondary causes [15]. Similarly, in our tertiary children's hospital, a wide PTC work-up protocol is conducted. This assessment causes blood loss, pain and discomfort, and a non-negligible financial burden.

To our knowledge, the rate of significant laboratory findings in the PTC work-up has not been studied, and its clinical relevance has not been assessed. Therefore, we aimed to investigate the clinical yield of PTC laboratory work-up and rate of secondary PTC detection in the pediatric population in a tertiary-care medical center.

## Materials and methods

The study was approved by our medical center's Ethical Board committee, in accordance with the amended principals of the Helsinki Accords. The study received a waiver regarding the need for informed consent.

### Design and population

Data were retrieved in this retrospective cohort study from medical records at Schneider Children's Medical Center of Israel, a university-affiliated tertiary hospital. Included were patients hospitalized for the first time during 2010–2020 and subsequently discharged with a definite diagnosis of PTC according to the modified Dandy criteria (before 2014) or Friedman's revised criteria (after 2014), as confirmed by a certified pediatrician and ophthalmologist.

All children underwent a comprehensive neurological examination, including assessment of cranial nerves, motor and sensory function, reflexes, and cerebellar signs, performed by a certified senior pediatrician. Most were also examined by a board-certified pediatric neurologist. A certified ophthalmologist performed a fundoscopic examination and visual fields testing in all patient. In addition, all children underwent brain imaging (CT and/or MRI), a LP with opening pressure measurement under sedation, and extensive laboratory work-up to identify a possible PTC etiology.

**Fig. 1:**
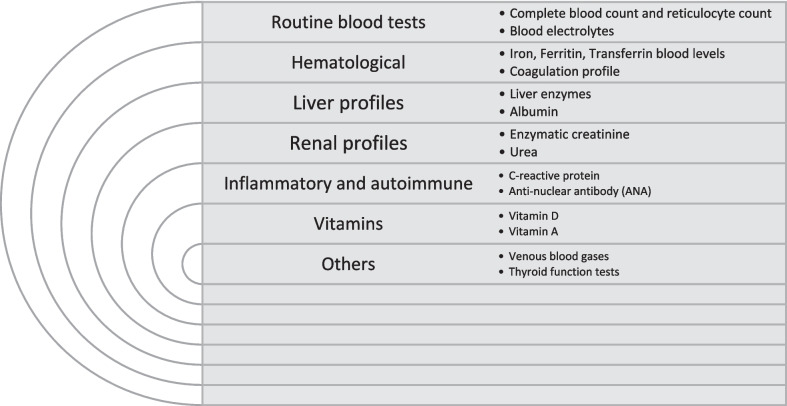
Laboratory workup in SCMCI

Figure 1 shows the routine laboratory work-up used during the study. All abnormal results were reviewed by two certified pediatricians to determine clinical significance. The etiologies for secondary PTC were sub-divided into two main groups-"definite secondary", for etiologies known in the current literature for PTC, and"probable association", for etiologies for which the association with PTC are less established or not evidence based.

Cut-off levels that define Vitamin D deficiency or Vitamin D insufficiency are controversial [16, 17]. We defined Vitamin D insufficiency as below 50 nmol/L and Vitamin D deficiency below 30 nmol/L, as recommended by The European Society of Pediatric Gastroenterology, Hepatology, and Nutrition, and by the American Institute of Medicine [18, 19].

Headache severity was routinely assessed using Visual Analog Scale (VAS) [20] by nursing staff upon admission and during each shift, in accordance with standard ward practice. VAS scores range from 0 (no pain) to 10 (worst pain). For this study, the highest VAS score on the first day (prior to the LP procedure) and during the entire hospitalization were retrospectively retrieved.

### Statistical analysis

Data were analyzed using IBM® SPSS software for Windows, version 25 (IBM Corp, Armonk, New York, USA). Continuous variables were expressed as mean ± SD if normally distributed or median [interquartile range] if non-normally distributed. Discrete variables were described as number (% of total).

## Results

The 75 children with a definite diagnosis of PTC (44 girls, 58.7%) included in the study had a mean age of 10.9 ± 4.25 years. The most common presenting symptom was headache, reported in 53/74 (71.6%) of patients. Among patients who reported headache at presentation, the median highest VAS score at admission was 3 (IQR 0–6), while the median highest VAS score during entire hospitalization was 6 (IQR 5–9). Moreover, diplopia or strabismus was reported in 14/73 (19.2%), blurred vision 16/68 (23.5) and vomiting in 16/73 (21.9%). More clinical data on the cohort is presented in Table 1. Mean BMI was 24.53 ± 8.65 kg/m^2^, 27/57 (47%) of the subjects were overweight (BMI > 25), and 18/57 (31%) were obese with BMI > 30. Secondary PTC (other than obesity) was diagnosed in 36 (48%) children. A definite secondary PTC was diagnosed in 15/75 (20%) children, of whom 8 (53.3%) were overweight and 3 (20%) obese.

**Table 1:** Characteristics of study group

**Variable**	Primary PTC n = 39	Secondary PTC n = 15	Probable secondary PTC n = 21	Total n = 75
Female gender (N (%))	26 (66.67%)	7 (46.67%)	11 (52.38%)	44 (58.7%)
Age (Years, mean ± SD)	11.35 ± 4.02	9.6 ± 4.7	11.05 ± 4.35	10.92 ± 4.22
Jewish origin (N (%))	27 (69.23%)	11 (73.33%)	20 (95.24%)	58 (77.3%)
Arabic origin (N (%)	12 (30.77%)	4 (26.67%)	1 (4.76%)	17 (22.7%)
Weight (kg, mean ± SD)	54.69 ± 26.33 n = 38	45.32 ± 27.45	55.32 ± 30.79 n = 20	52.94 ± 27.72 (n = 73)
Body Mass Index (Kg/m^2^,mean ± SD)	23.73 ± 8.98 n = 30	24.93 ± 7.33 n = 11	25.75 ± 9.22 n = 16	24.53 ± 8.65 (n = 57)
**PTC symptoms**				
Headache	29 (76.31%) n = 38	8 (53.33%)	16 (76.19%)	53 (71.6%) n = 74
Diplopia/strabismus	7 (18.42%) n = 38	3 (20%)	4 (19.05%)	14 (18.9%) n = 74
Tinnitus	1 (2.63%) n = 38	1 (6.67%)	1 (4.76%)	3 (4.1%) n = 74
Vomiting	8 (21.05%) n = 38	4 (26.67%)	4 (19.05%)	16 (21.6%) n = 74
Other	21 (60%) n = 35	7 (46.67%)	9 (47.37%) n = 19	37 (53.6%) n = 69
**Measurements**				
LP opening pressure (Mean ± SD)	39.38 ± 10.29 n = 38	39.9 ± 8.41	41.48 ± 11.66	40.08 ± 10.26 n = 74

The most common etiology of secondary PTC was treatment with PTC-causing medications. Four (4/15, 26.7%) patients were treated with Growth Hormone (GH), 3 (20%) had steroid treatment withdrawal and 2 (13.3%) had been using anti-acne medication. Other common etiologies are detailed in Table 2.

**Table 2:** Secondary causes for PTC in the study cohort

**Medication Related**	**N = 15**
GH therapy	4 (26.7%)
Steroid withdrawal/tapering	3 (20%)
Acne treatment (Isotrerinoin, Minocyclin)	2 (13.3%)
Anti-psychotic treatment (Risperidone	1 (6.7%)
Kidney disease*	4 (26.7%)
Known thyroid disease*	2 (13.3%)
Mastoiditis*	2 (13.3%)
**Sinusitis**	1 (6.7%)

^*^ Four patients had concomitant diseases—one chronic kidney disease (CKD), acute mastoiditis and epilepsy; one CKD and received growth hormone (GH) treatment; one steroid withdrawal due to the kidney disease; and one Duchene muscular dystrophy and steroid tapering and hypothyroidism

Of the 21 (21/75, 28%) patients with a pre-existing medical condition not known to cause PTC in the current medical literature, 5 (23.8%) were being medically treated for attention deficit hyperactive disorder (ADHD) and 3 (14.3%) for asthma or other chronic lung diseases, with 3 (14.3%) taking anti-seizure medications for epilepsy. 370795

Median follow-up time from admission to last documented visit in pediatric ophthalmology unit was 20.5 months, (IQR 10.75–38.25).

The etiological workup is outlined in Fig. 1. Mean White blood cell count (WBC) and hemoglobin level were within normal limits (9.04 ± 2.79 K/micL and 12.82 ± 1.15 g/dL, respectively). All had normal blood creatinine levels and normal liver function (except for one patient who had mildly elevated liver transaminases, which resolved spontaneously in follow-up). None of the patients had clinically significant abnormal thyroid function except two with known autoimmune hypothyroidism/thyroiditis prior to PTC diagnosis.

Vitamin D insufficiency below 50 nmol/L was diagnosed in 15/22 (68%) of the subjects and more than half of them had a vitamin D deficiency of less than 30 nmol/L. Vitamin A levels were normal in 91.6% (44/48) of patients, but the outliers (both above and below the normal limits) were mild and not clinically significant. One patient had laboratory tests compatible with antiphospholipid syndrome (APLA), without any clinical signs or symptoms during diagnosis or later in follow-up. None had any other clinically significant abnormal findings or needed additional tests or treatments.

## Discussion

In the present study, we found that secondary PTC is common in children, and accounts for at least one fifth of pediatric PTC cases.

All patients in this cohort underwent an extensive etiological work-up protocol. Other than vitamin D deficiency, we did not find other abnormal laboratory tests with proven clinical relevance to the diagnosis of PTC.

Although certain secondary causes in childhood are specific to the pediatric population, such as growth hormone replacement therapy or acne medications, we found a significant proportion of the secondary PTC cases in our cohort also exhibited a high prevalence of overweight or obesity. These findings are in line with previous reports. We hypothesize that this might represent a “double hit” phenomenon, in which some of the medical conditions and treatments served as inciting factors for developing PTC in children with a prior predisposition due to overweight or obesity [12].

Almost all cases of secondary PTC in our cohort were accurately identified by a thorough medical history and comprehensive physical examination. This observation agrees with a previously published cohort of secondary PTC among children and adolescents, where 16/75 patients with PTC had a definite diagnosis of secondary PTC. The etiologies were antibiotic treatments (11 patients), chronic kidney disease (3 patients), withdrawal from chronic glucocorticoid treatment (1 patient), and lithium treatment (1 patient) [12].

Vitamin D deficiency is not uncommon in the pediatric population and is more prevalent in obese patients. A meta-analysis of 20 studies and 4,600 children estimated the relative risk for the association between obesity and vitamin D deficiency as 1.41 [21]. Moreover, it seems that severity of obesity correlates with vitamin D blood level. Turer et al. found that the prevalence of vitamin D deficiency increased from 21% in normal- weight children to 49% in those severely obese [22]. It may be assumed that both the global obesity epidemic and high rates of vitamin D deficiency result from the sedentary lifestyle of children and adolescents in the western world, and may be a coincidental finding and not a direct cause for PTC.

During an 11-year period, except for vitamin D deficiency, we did not find clinically significant abnormal laboratory findings. Thus, we assume that even though the etiologies for secondary PTC may be different in the pediatric population, it might not be clinically justified to perform an extensive workup. Similarly, secondary etiologies with association to PTC, which are less familiar as a direct cause of PTC according to current literature, could be easily diagnosed via a thorough medical history and physical examination. Changing the approach for PTC etiology investigation protocol may prevent unnecessary and costly blood tests.

Our study has several limitations. First, due to its retrospective design, data were not consistently available for all patients. Second, the ability to assess a syndrome with moderately low incidence was constrained by the limited cohort size. Additionally, subgroup analyses of the secondary causes were based on very small numbers, reducing the statistical power and limiting the generalizability of the findings. However, in relation to current literature, this is a relatively large series, and the fact that none of the subjects had positive clinically significant findings at diagnosis and during follow-up (except vitamin D deficiency) supports the validity of our findings.

## Conclusion

Our findings raise the possibility that, in many cases, an extensive laboratory work-up—aside from vitamin D levels- may not be routinely necessary when a thorough medical history and physical examination are performed.

These findings should be corroborated in a larger, comparative, multicenter study.

## Data Availability

The data supporting the findings of this study are available from the corresponding author upon reasonable request.

## Abbreviations

**BMI**: Body mass index
**CSF**: Cerebrospinal fluid
**ICP**: Intracranial pressure
**IIH**: Idiopathic intracranial hypertension
**LP**: Lumbar puncture
**PTC**: Pseudotumor cerebri
